# Predictors of underachieved and overachieved fertility among women with completed fertility in Ghana

**DOI:** 10.1371/journal.pone.0250881

**Published:** 2021-06-11

**Authors:** Isaac Yeboah, Stephen Owusu Kwankye, Faustina Frempong-Ainguah

**Affiliations:** 1 Institute of Work, Employment and Society, University of Professional Studies, Accra, Ghana; 2 Regional Institute for Population Studies, University of Ghana, Accra, Ghana; University of Salamanca, SPAIN

## Abstract

**Background:**

A woman’s ability to achieve her preferred family size is critical in addressing issues of high fertility in sub-Saharan Africa. The socio-cultural context in sub-Saharan Africa presents some difficulty for the attainment of preferred fertility for many women. Few studies in sub-Saharan Africa have examined the extent to which women are unable to achieve their preferred family sizes. This study, therefore, examines the factors that are associated with the non-attainment of women’s preferred fertility by the end of their reproductive years.

**Data and methods:**

The study analyzed pooled cross-sectional data with a sub-sample of 1,888 currently married women aged 45–49 years from five rounds of the Ghana Demographic and Health Survey, 1993 to 2014. Test of associations and multinomial logistic regression analysis were used to examine the predictors of underachieved and overachieved fertility relative to achieved fertility.

**Results:**

The results indicate that 44 per cent of the women recorded overachieved fertility while about 36 per cent underachieved their fertility. Partner wants more, experiencing child loss and married more than once were significantly associated with overachieved fertility. Nonetheless, increased years of a woman’s education and delaying her at first birth were negatively associated with overachieved fertility. On the other hand, underachieved fertility was significantly associated with having a partner with fewer fertility preference, being of the Islamic faith and ever use of modern contraception.

**Conclusion:**

Partner’s fertility preference, child loss experience, marrying more than once and ever use of modern contraception were important predictors of a woman’s inability to achieve her fertility preference. Policies to regulate men’s fertility behaviour, delaying age at first birth, use of modern contraception, encouraging longer years of education, and reducing infant and child mortality are important strategies to achieve fertility preference in Ghana.

## Background

Fertility preference, which describes how many children a woman would want to have during her reproductive life, is lower in developed countries compared to that in developing countries [1, 2]. It has been documented that many women in developed countries are not able to achieve their low fertility preference leading to lower actual fertility [3, 4]. However, in developing countries, women’s average achieved fertility exceeds their high fertility preference resulting in a higher overall achieved fertility. Again, compared with women in other developing countries, a higher proportion of women in sub-Saharan Africa exceed their high fertility preference, thereby producing a much higher achieved fertility [2]. It is, therefore, not surprising that overachieved fertility (actual fertility being higher than fertility preference) is highest among women in sub-Saharan Africa (SSA) compared to their counterparts in other regions of the world [5, 6]. However, the concurrent presence of women with underachieved fertility can also be higher because of the high fertility aspirations they usually tend to have [5]. In this paper, we seek to examine what potential factors influence individuals to divert from their course of achieving their fertility preference by the time they complete their reproductive life course. We hypothesize that older women who have completed their reproductive cycle are likely to deviate from achieving their fertility preferences due to multiple marital experiences, child loss experiences and/or spousal fertility preference beyond their individual and environmental characteristics. Many of these women also tend to have unmet need for family planning especially for limiting births [7–9], thereby making them exceed their preferred fertility.

Our interest in investigating the observed fertility gaps of older women who have completed their childbearing cycle within the sub-region is influenced by the observed stalling of Ghana’s fertility rate in the recent past, which defies our understanding of the demographic transition theory and the resurgence of research interest in fertility desires, family size intentions and realization among various age groups across the globe in recent years [10–14]. However, the discussions seem to vary between developed and developing countries. Whereas in the developed countries the debate centres on the future demographic sustainability of their currently recorded below-replacement fertility levels due to underachieved fertility, that of the developing world is more on overachieved fertility [13, 14]. It has been observed that although fertility rate is also declining in the developing world, the pace is slowest in sub-Saharan Africa and the pattern is distinct compared to the pace and pattern in Latin America and Asia [15, 16]. Despite the diverging views and the components of the fertility change across the divide, several researchers are of the view that fertility preferences, fertility desires and intentions are very critical in our understanding of fertility transitions.

In Ghana, whilst there was rapid decline in fertility between 1988 and 1998, fertility was stalling between 2003 and 2014. Total fertility rate (TFR) declined from 6.4 in 1988 to 4.6 in 1998 [17–19], i.e., a reduction of 1.8 children during the period. TFR further declined from 4.4 in 2003 to 4.0 in 2008 but increased slightly to 4.2 in 2014 [19, 20]. This suggests that fertility increased by only 0.2 children during 2003–2008. Total fertility rate among currently married women also declined by nearly one child (0.9) from 5.5 in 1988 to 4.6 in 1998. On the other hand, fertility preference among the currently married reduced by 0.1 children between 2003 (4.8) and 2014 (4.7). This obviously shows a difference between what women prefer and what they achieve at the end of their reproductive life, resulting in an excess of actual fertility over preferred fertility in Ghana during the past decade.

Ghana has made significant progress in socio-economic development over the past decades. For example, 23 percent of the population above six years had never attended school in 2010, compared to 73 percent in 1960 [21]. The extent to which socio-economic development has interacted to influence fertility gap especially during the last two decades is, however, not clear. In largely patriarchal societies like Ghana, where traditional norms and value systems place on men more power in reproductive decision-making, it may be difficult for a woman to achieve her preferred fertility [22, 23] because of the influential position of men. For instance, a study in Ghana found that the highly educated young women with high fertility preference are not able to achieve their preferred fertility without the approval of their husbands [23].

Notwithstanding the cultural context of sub-Saharan African women that makes the attainment of a woman’s preferred fertility quite difficult, it is not so for all women. Yet, few studies have examined the factors that enable some women to actualize their preferred fertility in these socio-culturally high fertility settings in SSA. However, among the few studies that have examined differences between preferred and actual fertility, only few have simultaneously studied factors associated with underachieved and overachieved fertility among women who have completed their fertility [24–26].

In the light of the foregoing, this paper more specifically examines the relationship between fertility preference and actual fertility among women 45–49 years (who have completed their reproductive cycle) in Ghana, which is conceptualized as the fertility gap in the context of the child loss experiences of women. It is conceived that ordinarily, women with high fertility preference would accordingly have equally high actual fertility and women who indicate low fertility preference would correspondingly record low actual fertility with all things held constant. However, this reasoning is dependent on the biological capability of the women (and their male partners) to give birth in accordance with their preconceived fertility desire. At the same time, the realization of this desire could be affected by the child loss experiences of the women, which could shift a woman’s progression to her preferred fertility to be lower or higher, thereby creating a fertility gap between preferred and actual fertility. The limitation underlying this assumption is that the fertility preference question in the Ghana Demographic and Health Surveys (GDHS) is asked to women not before they began childbearing, but at their current ages at the time of the survey that brings in the issue of ex-post rationalization: women who already may have had more children at the time of the survey being more likely to rationalize their higher actual fertility by giving responses that would suggest a higher preferred fertility and vice versa.

Furthermore, we recognize that while for some women their fertility preference could remain fixed, for many others especially in the context of many developing countries, fertility preference could change in the course of childbearing [13, 14]. For example, in Malawi, women are reported to change their numeric preferences in response to their marital relationships (whether they become divorced, widowed or enter new marriage) or when their reproductive circumstances do change relative to pregnancies or child mortality [14, 15].

In the light of all these circumstances and limitations of the data being used for this study, we further hypothesize that women who would prefer lower fertility may end having higher actual fertility after experiencing child losses to mortality. Conversely, women who may prefer higher fertility may have lower actual fertility in the absence of child losses through deaths and/or having stable marital unions. In either of the two scenarios, a fertility gap is respectively created leading to overachieved fertility in the first instance and underachieved fertility in the second scenario. Sandwiched between these two are other women who would not experience instability in their fertility preferences irrespective of their child loss experiences, resulting in achieved fertility. Implied in these assumptions is that child loss experiences of women alongside other characteristics of the women and their partners could contribute towards our explanation of the fertility gap between preferred and actual fertility, a subject which has not attracted much research attention in Ghana.

The rationale for choosing women aged 45–49 years is that fertility gap provides a true reflection of the completed fertility of these women. This study contributes to the limited literature on fertility gap (differences between fertility preference and actual fertility) among women with completed fertility in sub-Saharan Africa by examining the predictors of underachieved and overachieved fertility in Ghana. Studying why women are unable to achieve their preferred fertility provides an opportunity to understand the unique characteristics associated with these women especially the role of child loss experiences of women which have implications for policy intervention.

## Theoretical framework

Various theoretical and conceptual frameworks have been postulated in studying fertility preferences, fertility intentions and fertility desires. These include the theory of planned behaviour [27], the classical rational choice model [28], and the life-span theory of control [29]. All these theories guided the selection of variables for this study. The theory of planned behaviour sees the occurrence of a behaviour as an individual’s intention to act in that way [27]. The theory identifies three factors that influence an individual’s intentions as perceived behavioural control, attitude toward behaviour and subjective norms. Although the theory is used in behavioural settings and not a fertility model, several studies have meaningfully applied the theory in the study of fertility behaviour [30–33].

This theory has been heavily criticized as considering fertility preference as being fixed or static [13, 16, 17]. However, since our focus in this study is not on dynamics and or fixed nature of fertility preferences, we draw on a modified component of the theory of planned behaviour to suit our purpose. The main postulation is that an individual’s actual fertility is, to a large extent, influenced by her fertility preference. For instance, women who prefer to have fewer children are expected to work towards achieving fewer children being born. The assumption of planned behaviour is that background factors influence fertility behaviour. However, there can be unforeseen constraints either biological, social, or physiological that could cause an individual to either underachieve or overachieve her fertility preference. It is further assumed that women’s socio-demographic characteristics and partner characteristics influence preferred fertility and actual fertility of women. Fertility preference could be influenced directly by socio-demographic characteristics including child loss, place of residence, education, ethnicity, religion, use of modern contraceptives, age at first birth and number of martial unions [32–34].

There are other women’s socio-economic and partner characteristics that could directly influence actual fertility either positively or negatively. These include rural-urban residential differences, education, ethnicity, religion, and number of marital unions [35, 36]. For instance, women residing in rural areas often tend to have higher actual fertility than their counterparts in urban areas. Similarly, apart from an individual’s socio-demographic factors that could influence fertility preference, their partners’ educational attainment, marital arrangement and their fertility preferences could affect actual fertility of women [37–39].

Factors that determine the difference between preferred fertility and actual fertility that is conceptualized as fertility gap in this paper include child loss experience, type of place of residence, years of schooling, ethnicity, religion, age at first birth, use of modern contraceptives, number of marital unions and partner characteristics such as partner’s educational attainment and fertility preference [5, 21]. Fertility gap is a relevant indicator of fertility because it evaluates whether a woman is able to achieve her preferred fertility or not. The gap between preferred and actual fertility can be reduced or widened when the woman’s individual and socio-demographic characteristics adjust to or are at variance with the partner’s characteristics. Women adjust their actual fertility in accordance with their partners’ preference [26, 27]. Thus, a woman whose husband or partner has higher education is more likely to have fewer children irrespective of her fertility preference. These are examined and guided by the planned behaviour framework to assess how individual and partner characteristics as well as women’s child loss experiences affect their attainment of achieved fertility, underachieved fertility or overachieved fertility using Ghana as a case study.

## Data and methodology

We sought permission and accessed the data from MEASURE DHS database at http://dhsprogram.com/data/available-datasets.cfm. The Ghana Demographic and Health Survey (GDHS) is a cross-sectional, nationally representative population-based survey of households, targeting the reproductive lives of women, age 15–49 years and men of 15–59 years.

The GDHS uses a two-stage sampling method made up of cluster sampling, followed by systematic sampling at the household within each cluster. The GDHS sample is, therefore, stratified according to the administrative regions as well as rural-urban classifications using a probability proportional to population size at the initial stage of selection. The study data are based on a subsample of women aged 45–49 years from the various rounds of the GDHS conducted between 1993 and 2014. Though Ghana has conducted six rounds of the demographic and health survey, that of 1988 was excluded as it did not have questions on spousal fertility preference, which was important for this analysis. The study pooled data from the five rounds of the GDHS for 1993, 1998, 2003, 2008 and 2014 drawing on the women’s dataset. Pooling together different cross-sectional surveys increased the sample size and resulted in an increase in the precision of parameter estimates which allow for more precise conclusions to be drawn especially on the sub-population of women age 45–49 years who have completed their reproductive lifespan and may not be sufficiently represented by a single round of survey for such analysis [40, 41].

The pooled dataset from the successive surveys was of great benefit to the study as each survey gives independent estimates of the same sets of measurement and relationships [42] and each year had all the needed variables for investigation. Although each single survey year dataset was weighted to correct or adjust for disproportionate sample selection and survey non-response [43], for the pooled multiple surveys dataset, the old single survey years’ weights were re-scaled by the total number of women age 45–49 years for all the survey years (target total) and divided by each year’s original total. A weighted pooled sample of 2,034 women, age 45–49 years and currently married, from the survey years between 1993 and 2014 was extracted. This number initially included all women who had never experienced any childbirth and child loss (31 cases) irrespective of whether they desired to have more children or not. These 31 childless women were not included in the multinomial regression since it would not be possible for them to have overachieved fertility, one of the outcomes of the model.

The dependent variable used in the analysis in this study is fertility gap. This was measured by subtracting children ever born from preferred fertility of the woman (number of children preferred). The GDHS measures fertility preference by asking women with children: “If you could go back to the time you did not have any children and could choose exactly the number of children to have in your whole life, how many would that be?” This question allows both numeric and non-numeric responses. The non-numeric responses include “Up to God,” and “Don’t Know”. Women who provided non-numeric responses (146 cases) were dropped from the study. The reason for the exclusion of non-numeric responses from the analysis is that studies suggest that women with numeric responses make predictive outcome of child preferences compared to those with non-numeric responses [44]. Besides, it is difficult to quantify how many children such responses might be equated to in determining how their actual fertility (which is known by number) could vary from their fertility preference, which in this case is without measurement and could also vary even among different women giving the same or similar responses. Therefore, a total weighted pooled sample of 1,888 currently married women, age 45–49, who have experienced childbirth and gave numeric response to the fertility preference question was used for the analysis.

The difference between preferred fertility and actual number of children ever born yielded three different outcomes: negative, zero and positive values. The negative values constituted overachieved fertility, the zero value was labelled as achieved fertility and the positive values greater than zero constituted underachieved fertility.

Both the socio-demographic profile of respondent and partner characteristics were examined as possible predictors of fertility gap. Child loss experience of a woman refers to women with children dying within 59 months after they are born either at infant or childhood ages. This variable is categorized into four: all children survived; one or more infant deaths; one or more child deaths; and both infant and child deaths. The other socio-demographic variables include age at first birth (categorized as under 18 years; 18–20 years; and 21 years and above); number of marital unions (once or two or more unions); ever-use of modern contraceptives (Yes or No); respondent’s educational attainment (in years); place of residence (urban or rural); religion (Catholic; Other Christian; Muslim; or Others) and ethnicity (Akan; Ga-Dangme; Ewe; Mole-Dagbani; or Others). The contraceptive schedule of the GDHS covers a range of questions including ever-use and current use of contraceptives. For this study, we did not focus on current use of contraceptives knowing that women within this age group have either ended or are ending their fertility. For this analysis, we selected ever-use of contraceptives to examine whether ever-use of contraceptives influences their achieved fertility and thereby contribute to explaining the variation between actual fertility and fertility preference which constitutes the fertility gap. Hence, it is expected that women who have ever used modern contraceptives may be more likely to have their actual fertility approximating their preferred fertility (i.e., achieved fertility) compared to those who never used modern contraceptives. Ethnicity was classified based on the lineage system in Ghana where the Akan ethnic group represents the matrilineage while Ga-Dangbe and Ewe to the south and Mole-Dagbani represent patrilineage. Partner characteristics include the partner’s level of education (in years) and his fertility preference (defined as both want same; partner wants more; partner wants fewer; and don’t know).

Descriptive techniques of analysis including the use of percentages were used to describe the differences among the women by fertility gap. Chi-square test (χ^2^) and one-way analysis of variance were used to test association between fertility gap and each of the explanatory variables at a p-value of p < .05. At the multivariate level, the multinomial logistic regression model was used to examine the net influence of possible individual socio-demographic and spousal characteristics on underachieved and overachieved fertility compared to achieved fertility among currently married women, age 45–49 years who have completed their reproductive cycle. Women with achieved fertility were used as the reference category because it was assumed that women’s actual fertility should equal their fertility preference in an ideal situation notwithstanding the fact that in sub-Saharan Africa the socio-cultural contexts present some difficulty for many women to attain their fertility preference. We, therefore, seek to understand what influences women to deviate from their expected fertility preference to realize either underachieved or overachieved fertility compared to others who are able to achieve their fertility preference. In other words, achieved fertility is considered as the reference group (base category) because it is considered as the ideal expectation although not the most frequent outcome of fertility gap in sub-Saharan Africa, and the two other outcome categories (underachieved and overachieved fertility) are estimated relative to achieved fertility. The model predicted underachieved and overachieved fertility among women who have ended their reproductive lives. The general multinomial logistic regression model that was used is as follows:

$$ln(\frac{Pr\left(Y=U\right)/X}{Pr\left(Y=A\right)/X})=\beta_{0}^{U}+\beta_{1}^{U}X_{1}+\cdots .+\beta_{n}^{U}X_{n}$$

$$ln(\frac{Pr\left(Y=O\right)/X}{Pr\left(Y=A\right)/X})=\beta_{0}^{O}+\beta_{1}^{O}X_{1}+\cdots .+\beta_{n}^{O}X_{n}$$

Since the preceding dependent variable has three categories, the model has two equations. The superscripts ‘u’ (underachieved fertility) and ‘o’ (overachieved fertility) in the equation indicate which outcome the parameters belong to. The equation for the log of the relative risk ratio has two equations and a constraint, where:

$X_{1}‐X_{n}$ are the predictor variables; and

β_o_, β_1_…β_n_ are regression coefficients.

The advantage of the multinomial is that it provides a way to explore the lack of symmetry between underachieved and overachieved fertility. At the multivariate level, using STATA, coefficients from the pooled data describe the relationship between the covariates and fertility gap.

## Limitations of the study

The main limitation is that the survey question on fertility preference derived from the desired family size question is prone to the challenge of ex-post rationalization. This is because respondents may not include possible child deaths in their ideal family size. In addition, preferred fertility is influenced by individual and household circumstances at the time of the survey. This could lead to respondents upwardly giving biased responses. Childhood mortality experience of women is one of the socio-economic variables in this study to address the problem of ex-post rationalization. Furthermore, findings from the survey could suffer from a time-sequence challenge in determining factors associated with fertility gap, hence, it is difficult to draw clear causality conclusions. Despite these limitations, this study is one of the few to examine the predictors of underachieved and overachieved fertility among women with completed fertility in the context of sub-Saharan Africa. Furthermore, the findings of this study provide policy makers with a clearer understanding of factors that need to be considered to further reduce fertility.

## Results

**Fig 1:**
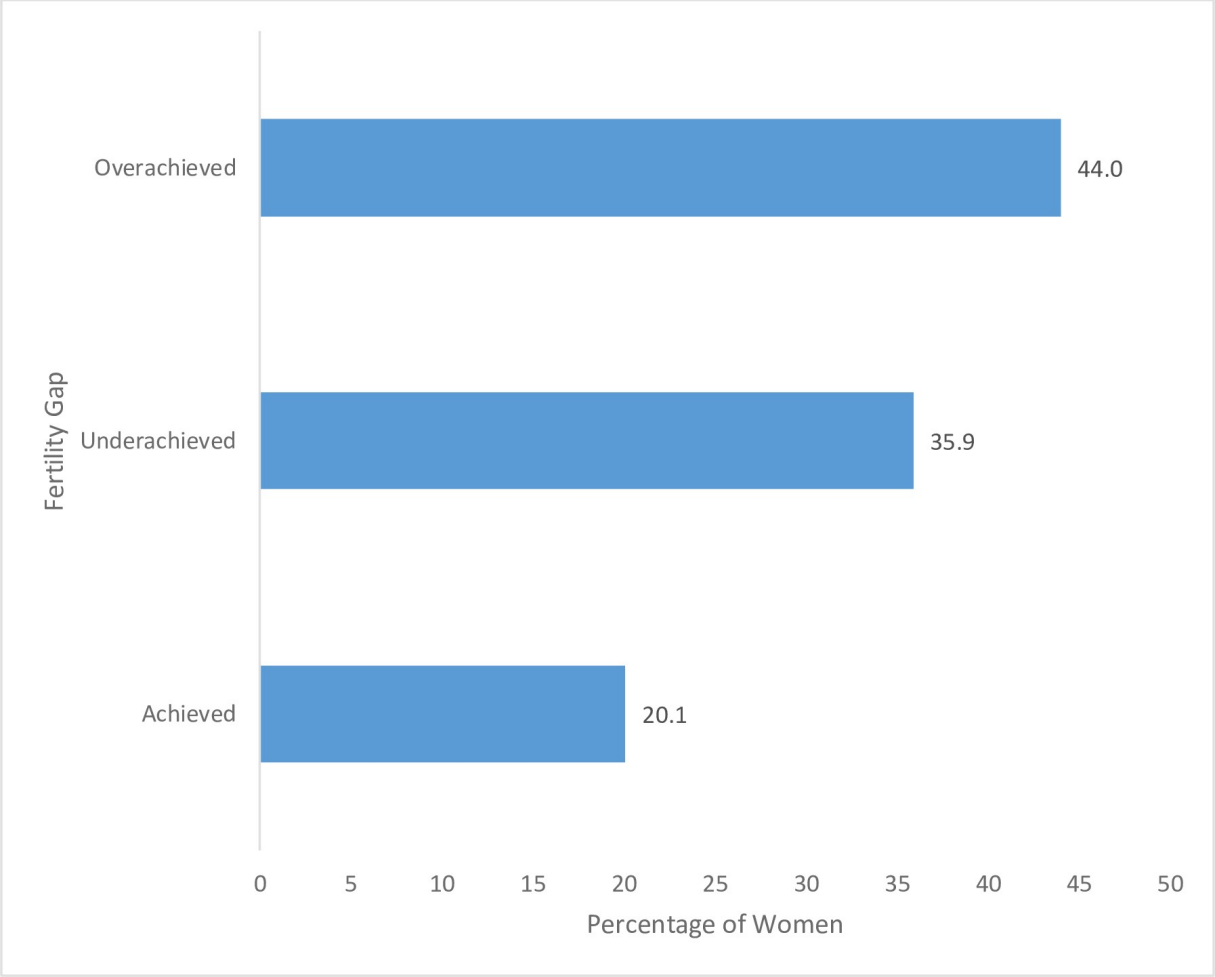
Percentage distribution of fertility gap among women with completed fertility in Ghana, 1993–2014. Source: Generated from GDHS 1993, 1998, 2003, 2008 &2014.

The results showed that of the 1,888 sub-sample of women, age 45–49 years, a higher proportion of women (44.0%) with completed fertility had recorded overachieved fertility, while 20.1% of them had achieved fertility with nearly 36 percent (35.9%) having underachieved fertility (Fig 1).

Women with higher mean number of children ever born reported having overachieved fertility (Table 1). There was statistically significant association between child loss experience and fertility gap. Underachieved and achieved fertility were higher among women who had all their children surviving beyond childhood (43.5% and 22.9% respectively). Overachieved fertility was higher among women who had an experience of both infant and child deaths. Underachieved and achieved fertility was significantly higher among urban residents than rural dwellers. In contrast, overachieved fertility was significantly higher among those who lived in rural areas compared to their urban counterparts (44.0% and 37.8% respectively). This means that there is need to focus increased policy attention to the rural areas in efforts towards addressing this observed “excess fertility” in the rural areas in comparison with the urban areas in the country. The results also show that education is significantly associated with achievement of fertility preference among the women studied. Thus, women with achieved fertility had higher mean years of schooling while their counterparts with the least mean years of schooling recorded overachieved fertility.

**Table 1:** Associations between independent variables and fertility gap in Ghana between 1993, 1998, 2003, 2008 & 2014.

Indicators	Underachieved Fertility	Achieved Fertility	Overachieved Fertility	F statistics/ Chi-Square
**Children ever born** Mean (SD)	4.6 (2.5)	5.7 (1.9)	7.5 (2.0)	F = 308.73***
**Child loss experience**				χ^2^ = 71.51***
All children survived	43.5	22.9	33.6	
One or more infant deaths	33.2	12.4	54.3	
One or more child deaths	37.0	19.0	43.9	
Both infant and child deaths	26.5	17.5	56.1	
**Place of residence**				
Urban	40.6	21.7	37.8	χ^2^ = 7.37*
Rural	37.5	18.4	44.0	
**Women Level of Education (Years of Schooling)** Mean (SD)	4.0(6.1)	4.5(5.4)	3.7(4.7)	(F = 3.03)*
**Ethnicity**				
Akan	32.0	19.7	48.3	χ^2^ = 51.56***
Ga-Dangme/ Ewe	34.1	18.3	47.6	
Mole-Dagbani	46.4	21.1	32.6	
Others	48.3	18.9	32.7	
**Religion**				
Catholic	31.8	18.6	49.6	χ^2^ = 19.20**
Other Christians	34.8	20.9	44.3	
Muslim	46.4	17.2	36.4	
Others	40.1	19.5	40.4	
**Age at first birth**				
< 18 years	15.8	26.3	57.8	χ^2^ = 98.56***
18–20 years	20.0	30.5	49.5	
21 years or older	22.8	45.5	31.6	
**Number of marital unions**				
Once	39.5	23.4	37.1	χ^2^ = 40.16***
Two or more unions	37.2	13.1	49.6	
**Ever use of modern contraceptives**				
No	43.5	19.3	37.2	χ^2^ = 52.53***
Yes	28.1	20.4	51.5	
**Partner’s Level of education (Years of schooling)** Mean SD)	5.6(6.0)	6.4(6.4)	6.2(5.8)	(F = 2.91)
**Couple’s fertility preference**				
Both want same	35.2	23.6	41.2	χ^2^ = 29.33***
Partner wants more	38.5	16.8	44.8	
Partner wants fewer	56.1	16.8	27.1	
Don’t know	40.4	16.4	43.2	
**Year of Survey**				
1993	24.8	14.3	60.9	χ^2^ = 70.25***
1998	34.9	14.0	51.0	
2003	32.2	21.8	45.9	
2008	35.0	23.7	41.2	
2014	43.8	22.3	33.8	

Source: Generated from the pooled sample data GDHS 1993, 1998, 2003, 2008 2014

* p < .05

**p < .01

*** p < .001.

Table 1 also shows that ethnicity was significantly associated with fertility gap. More specifically, achieved fertility was higher among women belonging to the Mole-Dagbani ethnic group compared to the other ethnic groups. Twenty-one percent (21.1%) of Mole-Dagbani women had achieved fertility at the end of their reproductive lives. In contrast, overachieved fertility was higher among women belonging to the Akan ethnic group and lowest among the other ethnic groups. Close to half (48.3%) of the Akan recorded overachieved fertility. Association between age at first birth and fertility gap was also statistically significant at p< 0.05.

Underachieved and achieved fertility was highest among women who had first birth at later ages (21 years and above). More than half (57.8%) of women who have completed their reproductive cycle with first birth before 18 years had overachieved fertility.

The number of marital unions was significantly associated with fertility gap. While underachieved and achieved fertility was highest among women who had been in one marital union (39.5% and 23.4% respectively), overachieved fertility was highest among women who had been in two or more unions.

Ever-use of modern contraceptives was significantly associated with fertility gap. The results in Table 1 show that underachieved fertility was highest among women who have never used modern contraceptives while achieved fertility was highest among women who have ever used modern contraceptives (43.5% and 20.4%). However, a higher proportion of women who have ever used modern contraceptives recorded overachieved fertility compared with women who have never used modern contraceptives (51.5% and 37.1% respectively).

Partner’s fertility preference was significantly related to fertility gap. Underachieved fertility was higher among women whose partners want fewer children compared to others whose partners desire to have more children. Concerning achieved fertility, almost a quarter of women (23.6%) whose fertility preference was the same as their partners were associated with achieved fertility. On the other hand, close to half (44.8%) of women whose partners desired higher fertility than them overachieved their fertility preference.

## Predictors of fertility gap

Table 2 indicates that likelihood ratio for the pooled dataset is significant at 95% with p-value less than 0.05, implying that the independent variables are jointly contributing to modeling the fertility gap. The results of the multinomial logistic regression model are presented in Table 2. The results show that child loss experience is a statistically significant predictor of fertility gap. Women who had experienced one or more infant deaths were 1.5 times more likely to have underachieved fertility (RRR = 1.51; p<0.05). This pattern is similar to the results on overachieved fertility where women who had one or more infant deaths were more likely to attain overachieved fertility.

**Table 2:** Results of multinomial logistic regression of fertility gap in Ghana between 1993, 1998, 2003, 2008 & 2014.

	Underachieved vs Achieved				Overachieved vs Achieved			
Indicators	RRR	S.E	P-value	95% C.I	RRR	S.E	P-value	95% C.I
**Child Loss Experience (**ref = All children survived)								
One or more infant deaths	1.51	0.30	0.04	[1.02, 2.23]	2.98	0.58	0.00	[2.03, 4.37]
One or more child deaths	0.88	0.17	0.50	[0.60, 1.28]	1.51	0.29	0.03	[1.04, 2.20]
Both infant and child deaths	0.69	0.18	0.16	[0.41, 1.15]	1.95	0.47	0.01	[1.22, 3.12]
**Level of education** (years in schooling)	1.01	0.01	0.42	[0.99, 1.04]	0.96	0.02	0.02	[0.93, 0.99]
**Place of Residence** (ref = urban)								
Rural	1.12	0.17	0.48	[0.82, 1.51]	1.24	0.19	0.18	[0.91, 1.68]
**Ethnicity** (ref = Akan)								
Ga-Dangme/Ewe	1.22	0.24	0.32	[0.83, 1.78]	1.12	0.21	0.55	[0.77, 1.62]
Mole-Dagbani	1.24	0.29	0.34	[0.79, 1.95]	0.50	0.12	0.00	[0.32, 0.80]
Others	1.52	0.34	0.06	[0.99, 2.35]	0.62	0.14	0.04	[0.39, 0.98]
**Religion** (ref = Catholic)								
Other Christians	1.10	0.23	0.64	[0.74, 1.65]	0.94	0.19	0.74	[0.63, 1.39]
Muslim	1.98	0.51	0.01	[1.20, 3.28]	1.18	0.32	0.53	[0.70, 1.99]
Other	1.16	0.28	0.53	[0.72, 1.87]	0.70	0.17	0.14	[0.44, 1.13]
**Age at First birth** (ref = <18 years)								
18–20 years	0.99	0.19	0.96	[0.68, 1.45]	0.85	0.16	0.36	[0.59, 1.21]
21 years+	1.28	0.23	0.18	[0.90, 1.83]	0.57	0.10	0.00	[0.40, 0.80]
**Number of marital unions** (ref = once)								
Two or more unions	1.87	0.29	0.00	[1.38, 2.53]	1.87	0.28	0.00	[1.38, 2.52]
**Ever use of modern contraceptive** (ref = No)								
Yes	0.63	0.09	0.00	[0.47, 0.83]	1.36	0.19	0.03	[1.03, 1.80]
**Partners Level of education** (Years of schooling)	0.99	0.02	0.95	[0.97, 1.03]	1.00	0.02	0.97	[0.97, 1.03]
**Couple’s fertility preference** (ref = Both want same)								
Partner wants more	1.37	0.25	0.09	[0.95, 1.96]	1.63	0.30	0.01	[1.14, 2.34]
Partner wants fewer	2.37	0.70	0.00	[1.33, 4.22]	0.75	0.24	0.37	[0.39, 1.41]
Don’t know	1.48	0.24	0.02	[1.08, 2.04]	1.39	0.23	0.05	[1.01, 1.91]
**Year of Survey** (ref = 1993)								
1998	1.53	0.44	0.15	[0.86, 2.70]	0.78	0.21	0.35	[0.46, 1.32]
2003	0.93	0.25	0.79	[0.55, 1.58]	0.52	0.13	0.01	[0.32, 0.84]
2008	1.21	0.33	0.50	[0.70, 2.06]	0.46	0.12	0.00	[0.28, 0.76]
2014	1.18	0.30	0.52	[0.72, 1.95]	0.38	0.09	0.00	[0.24, 0.61]
Log pseudolikelihood	-1738.38							
LR Chi2 (p-value)	323.810 (0.000)							

Abbreviations: RRR = Relative Risk Ratio; S.E. = (Standard Error); C.I. = (Confidence Interval); Source: Computed by author using the GDHS 1993, 1998, 2003, 2008, 2014 * p < .05; **p < .01; *** p < .001.

Education also emerged from the analysis as a statistically significant predictor of fertility gap where a woman with increased number of years of schooling were less likely to have attained overachieved fertility by 3.8% (RRR = 0.96; p<0.05) compared to achieved fertility. Thus, women with higher number of years in education were significantly less likely to have overachieved fertility by the end of their reproductive lives.

Regarding ethnicity, women belonging to the Mole-Dagbani ethnic group were less likely to have attained overachieved fertility compared to achieved fertility by 50% (RRR = 0.50; p<0.01). Similarly, women belonging to the Other ethnic group (predominantly ethnic groups in northern Ghana) were less likely to have attained overachieved fertility with respect to achieved fertility by 32%. This suggests that women belonging to patrilineal ethnic groups were significantly less likely to have overachieved fertility compared to achieved fertility.

Women who experienced first birth at the age of 21 years and above were less likely to attain overachieved fertility compared to achieved fertility by 43% (RRR = 0.57; p<0.01). This means that women who delayed first birth were significantly less likely to have overachieved fertility with respect to achieved fertility.

The number of marital unions of a woman was a statistically significant predictor of fertility gap. Women who had been in two or more marital unions were more likely to have underachieved fertility compared to achieved fertility by 1.9 times (RRR = 1.87; p<0.001). Similarly, those who had been in two or more marital unions were more likely to have overachieved fertility compared to achieved fertility by 1.9 times (RRR = 1.87; p<0.001).

It is to be noted that the results relative to the use of modern birth control methods were contrary to expectation. Women who have ever used modern contraceptives are more likely to have overachieved fertility compared to achieved fertility by 1.4 times (RRR = 1.36; p<0.05). This could indicate that women use contraception when they perceive a risk of overachieving fertility or have already overachieved fertility.

The results further show that partners’ fertility preference is a significant predictor of fertility gap. A woman with a partner who wants more children than her was 1.6 times more likely to have overachieved fertility compared to achieved fertility (RRR = 1.63; p<0.01). Again, a woman who does not know the partner’s preference was 1.4 times more likely to have overachieved fertility relative to achieved fertility (RRR = 1.39; p<0.05). The results from Table 2 also suggest a trend toward lower incidence of overachieved fertility. Table 2 shows a decline in the proportion of women who have attained overachieved fertility from 60.9% in 1993 to 33.8% in 2014. On the other hand, those attaining achieved fertility have increased from 14.3% in 1993 to 22.3% in 2014. The proportion of women that had underachieved fertility increased from 24.8% in 1993 to 43.8% in 2014.

## Discussion

Understanding the preferred and actual fertility nexus sheds light on why some women are unable to achieve their preferred fertility and why others are able to do so [24]. However, issues relating to the former have been ignored because the concerns of sub-Saharan African countries have mainly been why women tend to have higher fertility than desired. Nevertheless, recent studies show a large proportion of women either overachieving or underachieving their fertility preference. Studying why women attain underachieved or overachieved fertility provides an opportunity to understand the unique characteristics associated with these women which may have implications for policy intervention and population management.

The study examined the predictors of underachieved and overachieved fertility among currently married women aged 45–49 years in Ghana between 1993 and 2014. This study identified nine factors that predicted overachieved and underachieved fertility: child loss experience, education, ethnicity, religion, age at first birth, number of marital unions, ever-use of modern contraceptives; partner’s fertility preference and year of survey. Five factors predicted both underachieved and overachieved fertility: child loss experience, ethnicity, number of marital unions, ever-use of modern contraceptives and partner’s fertility preference.

The results indicate that a higher proportion of women who had all their children surviving child deaths achieved fertility. Achieved fertility was less likely to be attained among women with some child loss experience. This is consistent with what is found in the literature [24, 45]. This result suggests that child loss by a mother has important impacts on her decision to continue having additional children in Ghana. It is also possible that with the experience of child loss, couples may increase their preferred family size to ensure that the number of children they lose may not prevent them from reaching their desired fertility preference or they will have their desired number of children grow into adulthood [46, 47].

Regarding education, the study found that higher number of years of education increases the likelihood of underachieved fertility and reduces the likelihood of overachieved fertility. Several studies in sub-Saharan Africa have also identified education as an important panacea to reducing fertility or achieving a woman’s preferred fertility [5, 48]. Education improves knowledge on reproductive health, exposes women to new gender and family norms, increases access to the mass media that could influence people’s attitudes and knowledge on fertility [49, 50]. Education also tends to promote achieved or underachieved fertility by postponing women’s entry into first birth, suggesting that later age at first birth is associated with relatively many years of childbearing cut off and thereby reducing the likelihood of having overachieved fertility.

Research has shown that the likelihood of achieved fertility occurring is higher when first birth is delayed to later ages. This is expected because early age at first birth may predispose most women to subsequent births especially when the births push them into marriage where they may experience longer reproductive years, which could expose them to a higher risk of subsequent births. In contrast, later age at first birth will tend to shorten the reproductive years of women and reduce their fertility in the long run.

Furthermore, compared to being in one stable union, being in two or more unions increased the relative likelihood of having overachieved fertility than achieved fertility by 1.9 times. Similarly, being in two or more unions increased the likelihood of underachieved fertility than achieved fertility. This suggests that a woman who has been in more than one union in her reproductive life is significantly less likely to achieve her preferred fertility. The finding from this study is consistent with what some studies have shown [25, 51, 52]. A study conducted in Brazil by Carvalho et al. (2016) [25] showed that remarriage encourages new childbearing as a way of sealing marriage bonds which may contradict with the woman’s initial preferred fertility. The suggestion from the foregoing is that women in one continuous union are less likely to overachieve their fertility than their counterparts who have been in more than one union. Having ever been in more than one union probably increases the pressure to bear children as a means of security between the couples.

We, however, note from some other studies in sub-Saharan Africa that compared to women in one continuous union, women who have ever been in more than one union have lower fertility [53]. An explanation for this is that women in previous marriages characterized by childlessness are more likely to have fewer children compared to women in only one union. Thus, childlessness in previous marriages that might have accounted for their dissolution may cut shorter the active reproductive years of women in their second or higher-order marital unions and thereby be associated with relatively lower fertility.

The finding that ever-use of modern contraceptives predicted a less likelihood of underachieved fertility and more likelihood of overachieved fertility relative to achieved fertility is quite unexpected because contraceptive use is expected to reduce fertility among women who actively practise contraception. However, from the results presented in Table 1, a higher proportion of women with overachieved fertility had experienced both infant and child deaths. Therefore, despite using modern contraceptives, their experience of child loss may predispose them to have more children than they preferred. It is also possible that many of those who reported to have ever used modern contraceptives practised contraception after they had had more than their preferred number of children already. It may be plausible to further explain that modern contraceptives were used by these women to space their high preferred number of births rather than to limit births.

The results reported on partners’ fertility preference imply that men’s importance over reproductive decisions is critical in placing reduction in fertility preference and actual fertility in context [23, 54, 55]. These results indicate that fertility is a joint decision and that women whose partner desired more (or less) children than herself end up having more (or less) children. As DeRose & Ezeh [22] explain, despite the educational level of many women, they tend to defer to the preference of men to prevail in most fertility decisions especially in marital unions.

The clear trend toward lower incidence of overachieved fertility could be related to decreasing unmet need for family planning in sub-Saharan Africa. A Study by Nyarko et al [7] using the 2003, 2008 and 2014 Ghana Demographic and Health Survey data has shown that there is a declining proportion of women not using contraception when they don’t want to get pregnant.

## Conclusion

This study observes that underachieved and overachieved fertility is not uncommon among women with completed fertility in Ghana. Furthermore, we have shown that overachieved fertility is declining over the years as represented by the five rounds of the GDHS. This could be due to other factors not included in the regression model. Unmet need for family planning is one of them.

A partner’s preference for more children predicted an increased likelihood of overachieved fertility. Considering the role played by men in women’s ability to achieve their preferred family size, efforts to reduce fertility in sub-Saharan Africa must consider how to reduce men’s higher fertility preference. We, therefore, recommend that programmes to reduce fertility in sub-Saharan Africa must consider the significant role played by men in fertility decisions in sexual partnerships especially in marital unions. In addition, researchers must consider placing equal emphasis on the fertility behaviour of men in any analysis of fertility among women to be able to properly inform relevant policy interventions towards achieving further fertility reduction in Ghana and the rest of sub-Saharan Africa.

The study findings further suggest some research implications that indicate that reproductive goals are increasingly achieved; further research could assess the fertility implications of this important result. Prospective birth interval data could be used to examine the impact of the death of a child on subsequent birth intervals, thereby assessing replacement effects on fertility goals among women in Ghana and elsewhere in sub-Saharan Africa.

## References

[1] Baizan P, Arpino B, Delclòs CE. The effect of gender policies on fertility: The moderating role of education and normative context. Eur J Popul. 2016;32(1):1–30. doi: 10.1007/s10680-015-9356-y 27069290 PMC4803818

[2] Gunther I, Harttgen K. Desired fertility and children born across time and space. Courant Res Cent Poverty, Equity Growth-Discussion Pap. 2013;144:1–22.

[3] Adsera A. An economic analysis of the gap between desired and actual fertility: The case of Spain. Rev Econ Househ. 2006;4:75–95.

[4] Hagewen KJ, Morgan SP. Intended and ideal family size in the United States, 1970–2002. Popul Dev Rev. 2005;31(3):507–27. doi: 10.1111/j.1728-4457.2005.00081.x 20376334 PMC2849141

[5] Channon MD, Harper S. Educational differentials in the realisation of fertility intentions: Is sub-Saharan Africa different? PLoS One. 2019;14(7):1–14. doi: 10.1371/journal.pone.0219736 31318943 PMC6638943

[6] Casterline JB, Han S. Unrealized fertility: Fertility desires at the end of the reproductive career. Demogr Res. 2017;36(14):427–54.

[7] Nyarko S.H., Sparks C.S. & Bitew F. Spatio-temporal variations in unmet need for family planning in Ghana: 2003–2014. Genus (2019), 75, 22 10.1186/s41118-019-0069-7.

[8] Wulifan J. K., Mazalale J., Kambala C., Angko W., Asante J., Kpinpuo S., et al. (2019). Prevalence and determinants of unmet need for family planning among married women in Ghana-a multinomial logistic regression analysis of the GDHS, 2014. Contraception and reproductive medicine, 4(1), 1–14. doi: 10.1186/s40834-018-0083-8 30723547 PMC6352348

[9] Govindasamy P, Boadi E. A decade of unmet need for contraception in Ghana: Programmatic and policy implications. Calverton, Maryland: Macro International Inc. and National Population Council Secretariat [Ghana]. 2000. doi: 10.1107/s0108270199009257

[10] Bhrolcháin MN, Beaujouan É. Do people have reproductive goals? Constructive preferences and the discovery of desired family size. In Analytical family demography 2019 (pp. 27–56). Springer, Cham.

[11] Bongaarts J, Casterline J. Fertility transition: is sub-Saharan Africa different? Population and development review. 2013 Feb;38(Suppl 1):153. doi: 10.1111/j.1728-4457.2013.00557.x 24812439 PMC4011385

[12] Hayford SR, Agadjanian V. Spacing, stopping, or postponing? Fertility desires in a sub-Saharan setting. Demography. 2019 Apr 15;56(2):573–94. doi: 10.1007/s13524-018-0754-8 30652298 PMC6450704

[13] Liefbroer AC. Changes in family size intentions across young adulthood: A life-course perspective. European Journal of Population/Revue européenne de Démographie. 2009 Nov 1;25(4):363–86. doi: 10.1007/s10680-008-9173-7 20016795 PMC2791833

[14] Trinitapoli J. Yeatman, S. The flexibility of fertility preferences in a context of uncertainity. Population and Development Review. 2018; 44(1) 87–116. doi: 10.1111/padr.12114 29695890 PMC5900734

[15] Atake EH, Ali PG. Women’s empowerment and fertility preferences in high fertility countries in Sub-Saharan Africa. BMC Women’s Health. 2019 Dec 1;19(1):54. doi: 10.1186/s12905-019-0747-9 30953494 PMC6451210

[16] World Bank, Africa’s population boom: Will it mean disaster or economic and human development gains. 2015. Available from: https://www.worldbank.org/en/news/press-release/2015/10/22/

[17] Ghana Demographic and Health Survey 1988. Ghana Statistical Service (GSS) & Macro International (MI). Vol. Calverton, Calverton, Maryland. 1989.

[18] Ghana Demographic and Health Survey 1998. Ghana Statistical Service(GSS) & Macro International Inc. Vol. Calverton, Calverton, Maryland. 1999.

[19] Ghana Statistical Service, Ghana Health Service, ICF International. Ghana demographic health survey 2014. Rockville, Maryland:USA [Internet]. 2015; Available from: https://dhsprogram.com/pubs/pdf/FR307/FR307.pdf

[20] Ghana Demographic and Health Survey 2003. Ghana Statistical Service (GSS), Noguchi Memorial Institute for Medical Research (NMIMR) & Macro International (MI). Calverton, Maryland. 2004.

[21] Ghana Demographic and Health Survey 2008. Ghana Statistical Service, Ghana Health Service, ICF Macro [Internet]. 2009. Available from: http://www.dhsprogram.com/pubs/pdf/FR221/FR221[13Aug2012].pdf

[22] DeRose LF, Ezeh AC. Men’s influence on the onset and progress of fertility decline in Ghana, 1988–98. Popul Stud (NY). 2005;59(2):197–210. doi: 10.1080/00324720500099496 16096198

[23] Dodoo FN, Landewijk P Van. Men, women, and the fertility question in Sub-Saharan Africa: An example from Ghana. Afr Stud Rev. 1996;39(3):29–41.

[24] Ibisomi L, Gyimah S, Muindi K, Adjei J. Ideal versus actual: The contradiction in number of children born to nigerian women. J Biosoc Sci. 2011;43(2):233–45. doi: 10.1017/S0021932010000684 21205376

[25] Carvalho A de A, Wong L, Miranda-Ribeiro P. Discrepant fertility in Brazil: An analysis of women who have fewer children than desired (1996 and 2006). Rev Latinoam Poblac [Internet]. 2016;10(18):83–106. Available from: http://www.redalyc.org/pdf/3238/323849388005.pdf

[26] Muhoza DN, Broekhuis A, Hooimeijer P. Variations in desired family size and excess fertility in East Africa. Int J Popul Res. 2014;2014:1–11.

[27] Ajzen I. The theory of planned behavior. Organ Behav Hum Decis Process. 1991;50:179–211.

[28] Johnson-Hanks J. When the future decides- uncertainity and intentional action in comtemporary Cameroon. Current Anthropology 2005; 46(3): 363–385.

[29] Heckhausen J. Schulz R. A life-span theory of control. Psychological Review. 1995; 102(2): 284–304. doi: 10.1037/0033-295x.102.2.284 7740091

[30] Balbo N, Mills M. The influence of the family network on the realisation of fertility intentions. Vienna Yearb Popul Res. 2011;9(2011):179–205.

[31] Dommermuth L, Klobas J, Lappegård T. Realization of fertility intentions by different time frames. Adv Life Course Res. 2015;24:34–46. doi: 10.1016/j.alcr.2015.02.001 26047988

[32] Khatun T. Desired and actual fertility in Bangladesh: The role of mass media and social interactions. Int Inst Soc Stud Netherlands (Masters Thesis). 2011;

[33] Kuhnt AK, Trappe H. Channels of social influence on the realization of short-term fertility intentions in Germany. Adv Life Course Res [Internet]. 2016;27:16–29. Available from: 10.1016/j.alcr.2015.10.002

[34] Philipov D, Bernardi L. Concepts and operationalisation of reproductive decisions: Implementation in Austria, Germany and Switzerland. Comp Popul Stud [Internet]. 2011;36(2–3):531–72. Available from: http://www.comparativepopulationstudies.de/index.php/CPoS/article/view/78

[35] Dibaba B. Factors Influencing Desired Family Size among Residents of Assela Town Journal of Women ‘ s Health Care. 2016;5(6):4–11.

[36] Berrington A, Pattaro S. Educational differences in fertility desires, intentions and behaviour: A life course perspective. Adv Life Course Res [Internet]. 2014;21:10–27. Available from: doi: 10.1016/j.alcr.2013.12.003 26047539

[37] Feyisetan B, Casterline JB. Fertility Preferences and Contraceptive Change in Developing Countries. Source Int Fam Plan Perspect [Internet]. 2000;26(3):100–9. Available from: http://www.jstor.org/stable/2648298%5Cnhttp://about.jstor.org/terms

[38] Klomegah R. Socioeconomic factors relating to fertility: A Ghanaian level test of the contextual theory of fertility. Int Rev Mod Sociol. 1999;29(1):17–33.

[39] Kravdal Ø. Education and Fertility in Sub-Saharan Africa: Individual and Community Effects. Demography. 2002;39(2):233–50. doi: 10.1353/dem.2002.0017 12048950

[40] Beck N, Katz JN. What to do (and not to do) with time-series cross-section data. Am Polit Sci Rev. 2006;100(4):634–47.

[41] Raffalovich LE, Chung R. Models for pooled time-series cross-section data. Int J Conf Violence. 2014;8(1):209–21.

[42] Reda AA, Lindstrom D. Recent trends in the timing of first sex and marriage among young women in ethiopia. African Popul Stud. 2014;28(2):1157–70. doi: 10.11564/28-0-564 27011431 PMC4800999

[43] Bethlehem J. Weighting. In: Encylopedia of Survey Research Methods. Thousand Oaks: Sage Publications, Inc; 2011. p. 958–60.

[44] Bachan L, Frye M. The decline in non-numeric ideal family size: A cross-regional analysis [Internet]. Unpublished Manuscript, The Pennsylvania State University, State College, PA & University of California at Berkeley, Berkeley, CA. 2013. Available from: http://paa2013.princeton.edu/papers/132008

[45] Gyimah SO. Fertility Response to Childhood Mortality in Sub- Saharan with Emphasis on Ghana and Kenya. PSC Discuss Pap Ser [Internet]. 2002;16(2):1–25. Available from: http://ir.lib.uwo.ca/pscpapers/vol16/iss2/1/?utm_source=ir.lib.uwo.ca%2Fpscpapers%2Fvol16%2Fiss2%2F1&utm_medium=PDF&utm_campaign=PDFCoverPages

[46] Gyimah SO, Fernando R. Intentional replacement of dead children in sub-Saharan Africa: Evidence from Ghana and Kenya. Can Stud Popul. 2004;31(1):33–53.

[47] Reher DS, Sandström G, Sanz-Gimeno A, van Poppel FWA. Agency in fertility decisions in western Europe during the demographic transition: A Comparative Perspective. Demography. 2017;54(1):3–22. doi: 10.1007/s13524-016-0536-0 28070854 PMC5306245

[48] Behrman JA. Does schooling affect women’s desired fertility? Evidence from Malawi, Uganda, and Ethiopia. Demography. 2015;52(3):787–809. doi: 10.1007/s13524-015-0392-3 25951799

[49] Bankole A, Ahmed FH, Neema S, Ouedraogo C, Konyani S. Knowledge of correct condom use and consistency of use among adolescents in four countries in sub-Saharan Africa. African J Reprod Heal [Internet]. 2007;11(3):197–220. Available from: http://www.ncbi.nlm.nih.gov/pubmed/18458741%0Ahttp://www.pubmedcentral.nih.gov/articlerender.fcgi?artid=PMC2367135 18458741 PMC2367135

[50] Dewi RRK, Suryadarma D, Suryahadi A. The Impact of Media on Behaviour: Evidence from Television Coverage Expansion and Declining Fertility in Indonesia. Dev Policy Rev. 2018;36:0552–63.

[51] Thomson E. Step-families and childbearing desires in Europe. Demogr Res. 2004;10:117–34.

[52] Thomson E, Lappegård T, Carlson M, Evans A, Gray E. Childbearing across partnerships in Australia, the United States, Norway, and Sweden. Demography. 2014;51:485–508. doi: 10.1007/s13524-013-0273-6 24399143 PMC5513161

[53] Tilson D, Larsen U. Divorce in Ethiopia: The impact of early marriage and childlessness. J Biosoc Sci. 2000;32:355–72. doi: 10.1017/s0021932000003552 10979229

[54] Caldwell J, Caldwell P. The cultural context of high fertility in sub-Saharan Africa. Popul Dev Rev. 1987;13(3):409–37.

[55] Dodoo FN. Relative spousal status and child health in sub-Saharan Africa: The case of Ghana. Sociol Q. 1994;35(3):507–19.

